# Projections Onto Order Simplexes and Isotonic Regression

**DOI:** 10.6028/jres.111.011

**Published:** 2006-04-01

**Authors:** Anthony J. Kearsley

**Affiliations:** Mathematical and Computational Science Division, National Institute of Standards and Technology, Gaithersburg, MD 20899

**Keywords:** isotonic regression, optimization, projection, simplex

## Abstract

Isotonic regression is the problem of fitting data to order constraints. This problem can be solved numerically in an efficient way by successive projections onto order simplex constraints. An algorithm for solving the isotonic regression using successive projections onto order simplex constraints was originally suggested and analyzed by Grotzinger and Witzgall. This algorithm has been employed repeatedly in a wide variety of applications. In this paper we briefly discuss the isotonic regression problem and its solution by the Grotzinger-Witzgall method. We demonstrate that this algorithm can be appropriately modified to run on a parallel computer with substantial speed-up. Finally we illustrate how it can be used to pre-process mass spectral data for automatic high throughput analysis.

## 1. Introduction

Given a finite set of real numbers, *Y* = {*y*_1_, …, *y*_n_}, the problem of isotonic regression with respect to a complete order is the following quadratic programming problem:

$$\begin{array}{l} {\text{minimize}}_{x}\;\sum_{i=1}^{n}w_{i}{\left(x_{i}-y_{i}\right)}^{2}\\ \text{subject to}\;x_{1}\leq \ldots \leq x_{n}, \end{array}$$ (1)

where the *w_i_* are strictly positive weights. Many important problems in statistics and other disciplines can be posed as isotonic regression problems. In epidemiology, binary longitudinal data are often collected in clinical trials of chronic diseases when interest is on assessing the effect of a treatment over time. Transitional models for longitudinal binary data subject to non-ignorable missing data can be developed but parameter estimation must be done in conjunction with an isotonic regression (see Ref. [3]). In classical time series analysis applied to global climate prediction, complex processes are often modelled as three additive components: long-time trend, seasonal effect and background noise. The long-time trend superimposed with the seasonal effect constitute the mean part of the process. The important issue of mean stationarity is usually the first step for statistical inference. Researchers have developed a testing and estimation theory for the existence of a monotonic trend and the identification of important seasonal effects. The associated statistical inference and probabilistic diagnostics result from solving a problem generically called the *change-point* problem. This change-point problem initially arose in quality control assessment. It includes, for example, the testing for changes in weather patterns and disease rates. Isotonic regression is necessary to test and estimate the trend and to determine periodic components (see Ref. [15]). For this application the isotonic regression yields estimators for the long-time trend with negligible influence from the seasonal effect, a desirable property. Recently, the development of algorithms for automatic peak and trough detection in raw mass spectrometer data have become an active research area. Motivated by the increased ability to produce high-quality data quickly, these algorithms have become crucial in cost-effective analysis of data (see Ref. [12, 13]). One difficulty in applying automatic structure revealing algorithms to raw spectroscopy data is the failure of data to be monotonic (usually as a function of mass or time). This can occur for many reasons including truncation and roundoff error, machine calibration errors, …etc. In this case, isotonic regression has become an important pre-processing step before data analysis.

Clearly the isotonic regression problem is an optimization problem encountered frequently when dealing with data generated with any uncertainty as is the case in many application problems in science and engineering. In their respective monographs Barlow, Bartholomew, Bremner, and Brunk and Robertson, Wright, and Dykstra have written comprehensive surveys of this subject (see Refs. [1, 9]).

### 1.1 A Simple Example

In this section we describe a simple example of the isotonic regression problem. Suppose that {1, 3, 2, 4, 5, 7, 6, 8} is a given set of real numbers, and that all the weights associated with these numbers are all identically one. This set is almost isotonic; however, {3,2} and {7,6} violate the nondecreasing requirement. One simple solution to this difficulty can be constructed by replacing each “block” of “violators” with the average of the numbers in the block. This produces {1, 2.5, 2.5, 4, 5, 6.5, 6.5, 8}, which turns out to be the unique solution of the isotonic regression problem. This is an example of the well-known “*Pool Adjacent Violators*” algorithm. One of the best-known examples of a pool adjacent violators algorithm for solving the isotonic regression problem is due to Grotzinger and Witzgall (Ref. [4]). In this important paper, the authors propose an efficient pool adjacent violator method for identifying data points that violate the order constraint and, in turn, projects the violators onto simplex representations of the order constraints.

In this work, the formulation of the Pool Adjacent Violators algorithm due to Grotzinger and Witzgall has been altered slightly to run specifically on parallel computers. This algorithm has been implemented and tested on parallel machines (Ref. [7]).

## 2. A Decomposition Theorem

To attain the necessary rigor, we exploit a famous and very elegant characterization of the solution to the isotonic regression problem. $W_{j}=\sum_{i=1}^{j}w_{j}$, let *P*_0_ denote the point (0, 0), and let *P_j_* denote the point (*W_j_*, $\sum_{i=1}^{j}w_{i}y_{i}$), for *j* =1, …, *n*. We interpret *P*_0_, …, *P_n_* as points in the graph of a function, which we extend to the interval [0, *W_n_*] by linear interpolation. Both the function and its graph are called the c*umulative sum diagram* (*CSD*) of the isotonic regression problem.

The *greatest convex minorant* (*GCM*) of a function *f* is the convex function defined by

GCM[f]=sup{ϕ:ϕconvex,ϕ≤f}.

It is a well-known and beautiful result that the isotonic regression problem is solved by taking $x_{j}^{*}$ to be the left derivative of *GCM* [*CSD*] at *W_j_*. Thus, theorems about isotonic regressions can be stated and proved as theorems about greatest convex minorants.

The ideas of the work presented here are based on convex analysis. A comprehensive survey of convex analysis can be found in Ref. [10]. Even though only elementary tools need be employed to prove this theorem, it has profound implications for parallel computation. Suppose that we decompose the set Y into *Y*_1_ ⊕ *Y*_2_, where *Y*_1_ = {*y*_1_, …, *y_k_*} and *Y*_2_ = {*y_k_* + 1, …, *y_n_*}. Analogously, we can decompose a function *f* with domain [0,*W_n_*] into *f*_1_ ⊕ *f*_2_, where *f*_1_ is the restriction of *f* to [0,*W_k_*] and *f*_2_ is the restriction of *f* to (*W_k_*, *W_n_*]. Then the following result is easily demonstrated.

**Theorem 1** *GCM* [*GCM*[*f*_1_] ⊕ *GCM*[*f*_2_]] = *GCM*[*f*]

Proof:

$$\begin{array}{l} \text{Since}\;GCM\left[f_{1}\right]\leq f_{1}\;\text{and}\;GCM\left[f_{2}\right]\leq f_{2},\\ \;GCM\left[f_{1}\right]⊕GCM\left[f_{2}\right]\leq f_{1}⊕f_{2}=f. \end{array}$$

It follows that, if *ϕ* ≤ *GCM*[*f*_1_] ⊕ *GCM*[*f*_2_], then *ϕ* ≤ *f*, and hence that

$$GCM\left[GCM\left[f_{1}\right]⊕GCM\left[f_{2}\right]\right]\leq GCM\left[f\right].$$

Conversely, suppose that *ϕ* ≤ *f* is convex and write *ϕ* = *ϕ*_1_ ⊕ *ϕ*_2_. Then *ϕ*_1_ ≤ *f*_1_ and *ϕ*_2_ ≤ *f*_2_, so *ϕ*_1_ ≤ *GCM*[*f*_1_] and *ϕ*_2_ ≤ *GCM*[*f*_2_]. It follows that *ϕ* ≤ *GCM*[*f*_1_] ⊕ *GCM*[*f*_2_], and hence that

$$GCM\left[f\right]\leq GCM\left[GCM\left[f_{1}\right]⊕GCM\left[f_{1}\right]\right].$$ (3)

Combining inequalities (2) and (3) gives the desired result. □

### 2.1 Implications for Parallel Computation

If one takes the function *f* to be the *CSD* for the isotonic regression problem, then Theorem 1 states the following: decomposing *Y* into *Y*_1_ ⊕ *Y*_2_, performing separate isotonic regressions on *Y*_1_ and *Y*_2_, and then, performing a final isotonic regression on the combined result, produces the isotonic regression on *Y*. Because the separate isotonic regressions on *Y*_1_ and *Y*_2_ can be performed simultaneously, parallel computations of isotonic regressions will be desirable if the final isotonic regression on the combined result is easy to compute. In point of fact, this is the case. Suppose that *Y*_1_ satisfies *y*_1_ ≤ ⋯· ≤ *y_k_* and *Y*_2_ satisfies *y_k_*_+1_ ≤ ⋯· ≤ *y_n_*. If *y_k_* ≤ *y_k_*_+1_, then *Y* is isotonic. If *Y* is not isotonic, then it must be because some of the largest numbers in *Y*_1_ exceed some of the smallest numbers in *Y*_2_. The antidote to this difficulty is to identify this central block of offending numbers and to replace each of these numbers with the weighted average of the block. (This is just the Pool Adjacent Violators algorithm again.) To accomplish this, let

$$\begin{array}{l} m=\min\left\{i:y_{i}>y_{k+1}\right\},\\ M=\max\left\{i:y_{i}<y_{k}\right\}, \end{array}$$

and

$$\bar{y}=\sum_{i=m}^{M}w_{i}y_{i}/\sum_{i=m}^{M}w_{i}.$$

Then, replacing *y_i_* with $\bar{y}$ for *i* = *m*,…, *M* gives the isotonic regression of *Y*. Thus, if one decomposes the isotonic regression problem and performs two smaller, separate isotonic regressions, it becomes fairly simple to obtain the solution to the original problem.

By now it should be apparent that what is being proposed in this paper is not a new parallel algorithm for isotonic regression that will compete with existing algorithms. Rather, it is the isotonic regression problem itself that has been parallelized. (An instructive analogy is the familiar exercise of sorting a list of numbers by subdividing the list, sorting each sublist, then interweaving the sorted sublists.) Because the problem itself has been parallelized, any isotonic regression algorithm can be used to compute the separate isotonic regressions assigned to separate processors. The efficiency of various isotonic regression algorithms has been discussed by Best and Chakravarti [2]. A very fast formulation of the Pool Adjacent Violators algorithm was provided by Grotzinger and Witzgall [4].

In light of the preceding arguments, we are virtually assured that a parallel approach to isotonic regression will speed up computation when *n* is sufficiently large. This phenomenon is demonstrated in Sect. 4. Notice, however, that we should not expect that the most efficient strategy will necessarily be the one that uses the largest number of processors, since the more that the original problem is decomposed, the more difficult it becomes to obtain the final solution from the separate isotonic regressions. As an extreme example of this limitation, one might decompose *Y* into *n* subsets of singleton values, in which case nothing whatsoever has been accomplished. Furthermore, the more that the original problem is decomposed, the greater the communication costs of parallelization. Hence, it is impossible to anticipate the most efficient decomposition strategy.

## 3. Application to the Analysis of Mass Spectral Data

Modern mass spectrometers are capable of producing large, high-quality data sets in brief periods of time (Ref. [14]). It is not uncommon for a synthetic polymer to produce a spectra with hundreds of peaks. This motivates the design of automated data analysis algorithms capable of rapid and repeatable processing of raw mass spectrometer data. While many algorithms for the analysis of raw mass spectrometer already exist, they all require significant operator input. In some cases smoothing parameters must be selected, in other cases one must identify peaks from noise or vice-versa, and many algorithms assume the functional form of data close to peaks or troughs. Once the data has been processed, for example peaks or troughs have been selected and the area underneath portions of the data have been calculated, there is still no standard or point of comparison (Refs. [5, 6]).

Recently an algorithm with the potential to automatically identify peak structure from raw mass spectrometer output without the use of smoothing, parameter specific filtering, or manual data analysis has been developed (Refs. [12, 8]). This method requires no knowledge of peak shape and no pre- or post-processing of the data. Experience to date on *matrix-assisted laser desorption/ionisation-time of flight mass spectrometry* (MALDI-TOFMS) shows that the power spectrum of the noise cannot be predicted solely from the experimental conditions; therefore, blind application of smoothing and/or filtering algorithms may unintentionally remove information from the data. The new method does not have this failing. It does not require equal spacing of data points. However, it does require that data be monotonic with respect to either mass or time. Because raw data may not be monotonic, because of machine error, rounding error, sample preparation … etc., an isotonic regression can and should be performed on raw data. The importance of being able to perform an isotonic regression without user-input is particularly important in this application. The goal of many of these algorithms is to provide output independent of *any* operator parameter selection or signal to noise estimation (Ref. [11]). In other words, the isotonic regression treatment of raw data must be extremely robust in order to be useful for this application.

## 4. Numerical Experiments

One of the largest potential consumers of these algorithms can be found in the field of *combinatorial chemistry*. Indeed, when one produces tens of thousands of mass spectra profiles, it is clear that sorting through each one of these profiles to identify peak and trough structure is simply not possible. As explained before, one cannot assume that the data will behave monotonically. For the purpose of this paper, we simulate this effect by taking the first component of ordered pair data produced from a MALDI-TOF mass spectrometer. This data is then corrupted in a way that introduces a simulated numerical error. This is very much like a combinatorial application where, for example, robotic schemes may be used to take mass spectral data from samples numerous times but automatically and rapidly.

The value *y_k_* is the *k*th first component of our unequally spaced data set of size *n*, where *n* = 50,000. The points are normalized so that *y_k_* ∈ [0, 100] for 1 ≤ *k* ≤ *n*. The resulting set *Y* of *n* increasing numbers was perturbed in various ways to obtain the data sets that we subjected to isotonic regression. Each of ten strategies for perturbing the original set of numbers was replicated *R* = 100 times, resulting in a total of one thousand data sets.

Let *σ* = log(2)/1.95996 be fixed. In what follows, whenever we perturb a value *y_k_*, we do so by replacing *y_k_* with *y_k_* exp(*σ z*), where *z* is a standard normal deviate. This multiplicative model of measurement error was constructed so that approximately 95 % of the perturbed values would be at least one half and no more than twice the replaced value.

The following loops describe our perturbation strategies. In each case, the intent was to perturb *P* values in the form of B blocks of length *L*.

For *R* = 1 to 100 repetitions:

1. Perturb *each* of the *n* values in the original data set *Y* to obtain data set Y1000.R.
2. For *P* = .49*n*, .25*n*, .01*n* and *L* = 1, $\sqrt{P}$, *P*:
  - Randomly select *B* = *P/L* numbers from {1, …, *n/L*} without replacement Call these numbers *s*_1_, …, *s_B_*.
  - Let *π* = *n/P*. For *i* = 1, …, *B* and j = 0, …, *L* − 1, let *k* = *πs_i_* + *j* and perturb each original value *y_k_*.
  - Denote the resulting data set by Y0ppl.R, where *pp* = 100*P/n* and *l* = 2 log (*L*)/log(*P*).
    For example, the data set produced on the fourth repetition of the case for which *P* = .49*n* and $L=\sqrt{P}$ is denoted by Y0491.4.

Thus, we generated 100 data sets (Y1000) in which all values were perturbed, 300 data sets (Y049l) in which 49 % of the values were perturbed, 300 data sets (Y025l) in which 25 % of the values were perturbed, and 300 data sets (Y001l) in which 1 % of the values were perturbed. Furthermore, in each of the cases that *P* = .49*n*, .25*n*, .01*n* of the values were perturbed, we generated 100 data sets (Y0pp0) in which we perturbed *P* isolated values, 100 sets (Y0pp1) in which we perturbed $\sqrt{P}$ blocks of $\sqrt{P}$ consecutive values, and 100 data sets (Y0pp2) in which we perturbed one block of *P* consecutive values.

Each data set was submitted to isotonic regressions on a personal computer cluster machine comprised of single and multiple processors. These regressions used, respectively, *A* = 1, 2, 4, 8, 16, 32 of the cluster computer’s processors. For each regression, the data set was decomposed into *A* subsets of (approximately) equal size. Each subset was simultaneously sent to a separate processor, where its isotonic regression was computed using Grotzinger’s and Witzgall’s [4] formulation of the Pool Adjacent Violators algorithm. As soon as the isotonic regressions of two consecutive subsets were computed, the combined result was sent to one of the available processors, which then computed the combined isotonic regression by means of the device described in Sec. 2.1. This process was continued until the isotonic regression of the entire data set was obtained. The elapsed time from job submission to completion was measured by the Delta’s intrinsic timer. The results are summarized in Table 1.

Table 1 exhibits several striking features. First, the variations in times produced by replications are extremely small relative to the magnitudes of the times. In retrospect this is not surprising: each data set contains a very large number of independent errors, so that one should expect that most data sets constructed in accordance with a specific perturbation strategy will be quite similar.

Second, there is very little variation in mean timing profiles between the perturbation strategies. This suggests that the phenomena described below are not unique to a particular data structure.

As anticipated, it is apparent that some degree of parallelization decreases the time required to perform an isotonic regression. For the data sets that we considered, the time required by *A* = 2 processors divided by the time required by *A* = 1 processor ranged from a minimum of 53.2 % to a maximum of 65.9 %, with a median of 60.3 %. The time required by *A* = 4 processors divided by the time required by *A* = 1 processor ranged from a minimum of 44.9 % to a maximum of 54.8 %, with a median of 50.1 %. The time required by *A* = 8 processors divided by the time required by *A* = 1 processor ranged from a minimum of 35.7 % to a maximum of 43.5 %, with a median of 40.5 %. Thus, there is compelling evidence that, for n = 50, 000 and these types of data sets, using *A* = 8 processors is more efficient than using *A* = 4, 2, 1.

For *A* = 16, 32, the communication costs of the parallelization strategy begin to dominate and the times are actually slower than for *A* = 8 processors. This phenomenon was also anticipated. With larger data sets, we know that we can take advantage of additional processors, but the tradeoff between *n* and the optimal *A* must be empirically determined for the data structures and parallel computing system of interest.

Finally we note that, although the proportional improvements in efficiency produced by parallel processing are impressive, the absolute times for serial processing are small. At present, it is difficult to forsee applications involving isotonic regressions on data sets so large that the absolute savings in time will warrant parallel computation. Perhaps that day will come; for now, our primary interest in parallelizing isotonic regression is for the pedagogical value of so doing. In our view, isotonic regression is a remarkably simple and elegant example of a problem for which mathematical theory virtually guarantees that parallelization will be beneficial.

## Figures and Tables

**Table 1:** Sample means and standard deviations ( $\bar{y}\pm s_{y}$) of elapsed times in milliseconds for five repetitions of ten isotonic regression experiments

Data Sets	Number of Processors					
	1	2	4	8	16	32
Y1000	2278± 93	1376±22	1158±16	930± 7	1062±25	1058±15
Y0490	2406±142	1416± 5	1182± 4	938± 8	1062± 4	1068± 8
Y0491	2376±180	1436±29	1208±50	958±19	1060±14	1080±27
Y0492	2370±171	1378± 8	1152± 8	922± 4	1032± 4	1036±15
Y0250	2396± 54	1386± 9	1144±11	944±48	1040±14	1040±12
Y0251	2298±128	1410± 7	1174± 5	936± 5	1058± 4	1052± 4
Y0252	2330± 95	1406± 9	1172± 4	942± 4	1066± 9	1058± 4
Y0010	2232± 30	1378± 4	1150± 7	922± 4	1034± 5	1032± 8
Y0011	2368±156	1410±10	1188±24	940± 0	1062± 4	1062± 4
Y0012	2380±152	1418± 8	1184±22	944± 5	1068±18	1070±12
